# Aging, Metabolism, and Cancer Development: from Peto’s Paradox to the Warburg Effect

**DOI:** 10.14336/AD.2017.0713

**Published:** 2017-10-01

**Authors:** Tia R. Tidwell, Kjetil Søreide, Hanne R. Hagland

**Affiliations:** ^1^Department of Mathematics and Natural Sciences, Centre for Organelle Research, University of Stavanger, Stavanger, Norway; ^2^Gastrointestinal Translational Research Unit, Molecular Laboratory, Hillevaåg, Stavanger University Hospital, Stavanger, Norway; ^3^Department of Gastrointestinal Surgery, Stavanger University Hospital, Stavanger, Norway; ^4^Department of Clinical Medicine, University of Bergen, Bergen, Norway

**Keywords:** Cancer, aging, mitochondria, metabolism, Warburg effect, Peto’s paradox, epigenetics

## Abstract

Medical advances made over the last century have increased our lifespan, but age-related diseases are a fundamental health burden worldwide. Aging is therefore a major risk factor for cardiovascular disease, cancer, diabetes, obesity, and neurodegenerative diseases, all increasing in prevalence. However, huge inter-individual variations in aging and disease risk exist, which cannot be explained by chronological age, but rather physiological age decline initiated even at young age due to lifestyle. At the heart of this lies the metabolic system and how this is regulated in each individual. Metabolic turnover of food to energy leads to accumulation of co-factors, byproducts, and certain proteins, which all influence gene expression through epigenetic regulation. How these epigenetic markers accumulate over time is now being investigated as the possible link between aging and many diseases, such as cancer. The relationship between metabolism and cancer was described as early as the late 1950s by Dr. Otto Warburg, before the identification of DNA and much earlier than our knowledge of epigenetics. However, when the stepwise gene mutation theory of cancer was presented, Warburg’s theories garnered little attention. Only in the last decade, with epigenetic discoveries, have Warburg’s data on the metabolic shift in cancers been brought back to life. The stepwise gene mutation theory fails to explain why large animals with more cells, do not have a greater cancer incidence than humans, known as Peto’s paradox. The resurgence of research into the Warburg effect has given us insight to what may explain Peto’s paradox. In this review, we discuss these connections and how age-related changes in metabolism are tightly linked to cancer development, which is further affected by lifestyle choices modulating the risk of aging and cancer through epigenetic control.

Human evolution has selected for somatic maintenance strategies that maximize reproductive success. However, the last century has provided us with a challenge where technology and lifestyle adjustments are outpacing natural evolutionary adaptation. Many of the previously life-shortening diseases, such as bacterial infections and viral diseases, can now effectively be treated, but other lifestyle-related diseases are increasing. Normal physiological responses are influenced by lifestyle habits such as high caloric diets, dysregulated sleep patterns, and toxic environmental factors; all common in modern Western civilization and known risk factors for developing cancer. The increase in life expectancy due to improved living standards and medical advances introduces the additional challenge of finding new treatments to treat the accumulation of age-associated diseases, including cancer.

As the aging population grows, cancer remains a fundamental health issue. Therefore, understanding its etiology and weaknesses to improve treatment is a major research focus worldwide. The lifetime risk of cancer has been associated with the number of stem cell divisions needed to maintain that tissue’s homeostasis, suggesting that acquired somatic nuclear mutations over time due to “bad luck” are the primary causes of cancers in these tissues. However, such a model fails to account for key observations of early-life mutation accumulation (50% before full body maturation) [1], the size and scaling of cancer incidence with the lifespans of various animals (Peto’s paradox) [2], and epidemiological research showing cancers of human populations differ based on geographical areas [3]. A more fitting model would be to ascribe the high incidence of cancers in tissues with more stem cell divisions to a buildup of bioenergetic dysfunction over time, which may confer a selective growth advantage in an aging tissue microenvironment. This could be affected, not only by inherited “bad luck” nuclear mutations, but more importantly, or external factors such as acquired epigenetic modifications, mtDNA mutations, or intrinsic asymmetric segregation of cargo during cell division.

## Aging and metabolic control

Aging is defined as a physiological decline that leads to the loss of major organ function, ultimately leading to death. The question remains as to what is the cause of aging as, from an evolutionary perspective, an organism would benefit most from extended reproductive ability and lifespan. A possible answer is that aging is but a side effect of life progression and not a programmed occurrence. Supporting this is the fact that rates of aging are not fixed; for example, lower body temperatures tend to result in extended lifespan [4], possibly due to metabolic adaptations. Considering the environmental effects on rates of aging, it has become increasingly relevant in aging research to differentiate between physiological age and chronological age [5]. It is possible for two humans of identical or similar years of life, or even same genetic background as with monozygotic twins, to have very divergent states of health and lifespans [6]. The last decade of scientific research has dramatically improved our understanding of the aging process and that it is closely regulated by key metabolic proteins such as mechanistic target of rapamycin (mTOR), AMP-activated protein kinase (AMPK), and insulin/insulin growth factor (IGF) [7, 8], which are associated with age-related metabolic syndrome [9] and common to those found dysregulated in cancer [10]. The long-term causative effect of aging and how this relates to increased cancer risk therefore seems to be linked through metabolic control.

**Figure 1.**
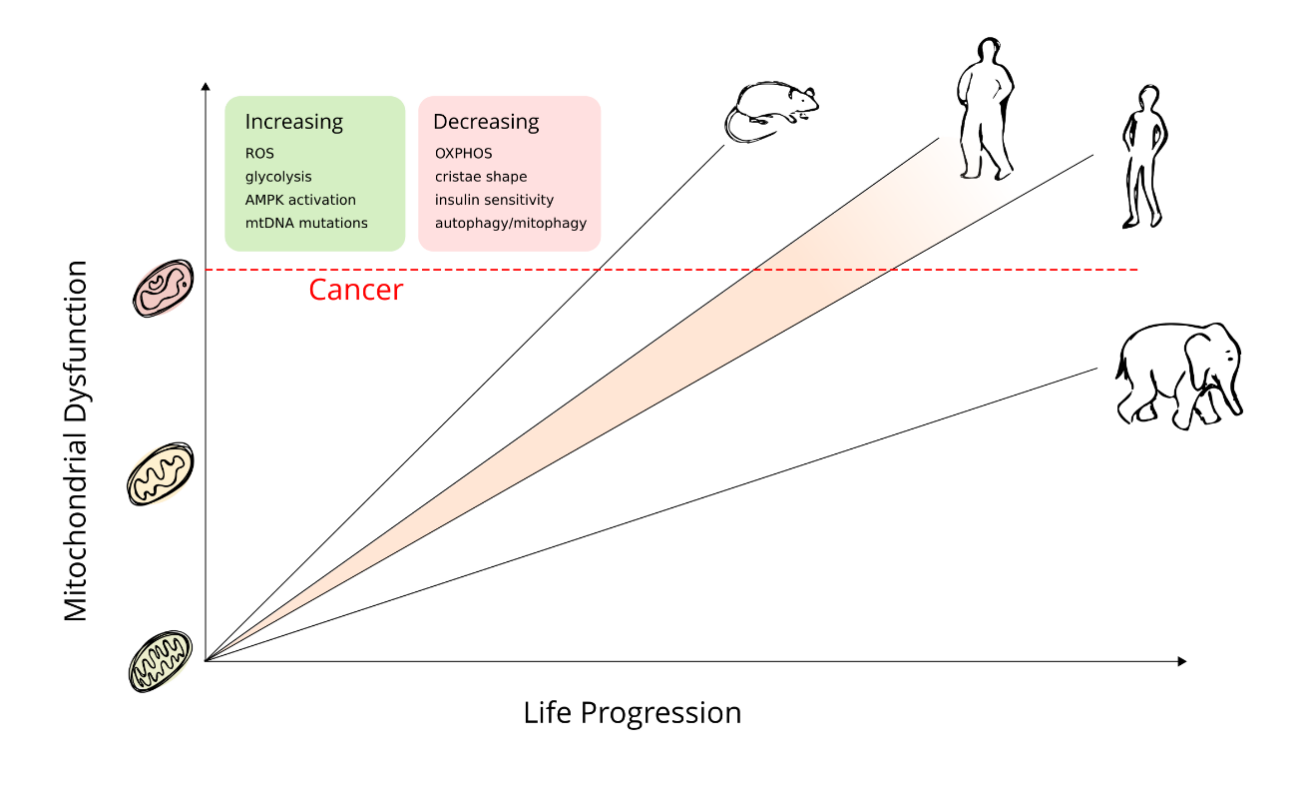
As animals age, there is an accumulation of dysfunction This affects the mitochondria to a great extent and a higher metabolic rate provides further amplification, reflected by the slope in this line. Once the dysfunction passes a threshold and the cell can no longer compensate, a cancerous transition may occur. The difference in resting metabolic rate (RMR) and their relative cancer development can be seen between large and small animals, with large animals having a low RMR and late or nonexistent cancer development. While RMR may not increase in larger individuals within species, metabolic stress accumulates at a faster rate and the individual can reach the dysfunctional threshold at an earlier timepoint, as exemplified here by the obese human figure having a shifted cancer risk.

Alternative to the mutational theory of cancer, the metabolic theory of cancer development is that small undetected changes in genes regulating metabolism, or mitochondrial genome mutations, can reach a threshold over time whereby it effects whole cell metabolism and confers a selective advantage for growth of that cell in an aging tissue environment [11]. Aging is linked to both the accumulation of genomic defects and that of defective proteins and organelles such as mitochondria [12, 13]. Consequently, mitochondrial defects can become prevalent in dividing stem cells by asymmetrical segregation of cell cargo. On the other hand, inheritance of “good” cargoes can enhance cell health and responsiveness, whereby more of the dysfunctional cargo is delivered to the daughter cell, which will go on to terminal differentiation, thus protecting the original stem cell [12]. Both internal and external factors may affect the growing number of dysfunctional mitochondria, speeding up the physiological aging process and cancer risk (Figure 1). However, while chronological age is immutable, physiological age depends on lifestyle choices and can be shifted to exert a beneficial effect by extending length of life and reducing disease risk.

Discoveries made in the last decade showing that most of the known oncogenes and tumor suppressor genes are metabolic regulators has rekindled Warburg’s discoveries made over a lifetime ago, highlighting the importance of metabolic control in any cell. The understanding that proteins and metabolites may be the instigators of aging and cancer development through epigenetic regulation is now a renewed research topic. This non-static mechanism of aging and cancer is gradually being accepted and helps explain why large long-lived animals with slow metabolism have a lower risk of developing cancer than humans. Of course, exposure to environmental factors are associated with increased cancer risk and can also contribute to changes in an aging system. However, these events will not be the primary focus here, since even in their complete absence, aging and cancer would occur. Therefore, this review focuses on the intrinsic events that may lead to aging and how they relate to cancer development, with a focus on the role of mitochondria and metabolism.

## Mitochondrial role in cell metabolism

Mitochondria are remnants of an aerobic prokaryote that brought the selective advantage of using respiration for higher yield energy production to a cell dependent on rudimentary substrate fermentation. The endosymbiotic event that occurred some two billion years ago is thought to only have happened once to give rise to all advanced lifeforms known today.

Mitochondria have retained some features of a prokaryote such as double membranes and their own DNA (mtDNA), which encodes for proteins and RNAs that are mostly involved in assembling components of the electron transport chain (ETC) [14]. Mitochondrial DNA mutations, deletions, and copy number changes can result in ETC dysfunction and are believed to accumulate with aging. The increase in dysfunction of energy homeostasis with age and increase in reactive oxygen species (ROS) has been the center of the free radical theory of aging [15]. Reactive oxygen species (ROS) have also been shown to affect ETC indirectly. In *C. elegans*, repression of the *PRDX-3* gene, involved in the detoxification of mitochondrial hydrogen peroxide in the ETC, did not alter the level of ROS or life length, but instead caused mitochondrial uncoupling and decreased adenosine triphosphate (ATP) production [16], suggesting an induced compensatory response. Continuous ROS exposure has been shown to affect mitochondrial oxidative phosphorylation (OXPHOS) and ATP production by lipid peroxidation of cardiolipins [17]. Cristae invaginations caused by the unique properties of cardiolipins is essential for efficient oxidative energy production and mitochondrial function [18, 19]. Defects in the cristae formation (i.e. lipid peroxidation due to ROS) can increase the leakiness of the inner mitochondrial membrane, consequently reducing the mitochondrial ATP production [20]. Alterations of cardiolipin through years of ROS exposure may lead to gradually reduced membrane potential and consequently depolarized mitochondria with less efficient ATP production. Another consequence is an imbalance in the metabolite levels in the aging cell which could affect gene regulation and transcription epigenetically. This nuclear-mitochondrial retrograde signaling, where gene expression is regulated by metabolic substrate levels, is important to respond appropriately to metabolic stress for restoration of cell homeostasis [21, 22]. One responder to this retro-grade signaling is the evolutionarily conserved human polymerase delta (*POLD1*) gene, which is involved in multiple forms of DNA repair, and found mutated in tumors and aging [23]. The expression of this protein is further dysregulated in diabetes [24] and can be modulated by enzymes involved in metabolism such as lactate dehydrogenase and 3-phosphoglycerate [25]. This retrograde signaling from mitochondria to nuclei is triggered in normal cells by changes in metabolite levels or altered proteostasis [22, 26, 27]. Response mechanisms can include upregulation of metabolic pathways producing more metabolites, including reactive oxygen species, to act as second messengers to tune signaling pathways in the cytoplasm or directly affect gene regulation through epigenetic events. Thus, the energy status of the cell is directly linked to its replicative and reparative functions, demonstrating how metabolic substrates and enzymes regulate cell turnover [28]. In support of dysfunctional metabolism controlling cell growth, laser capture of cancer cells from colon tissue, selected by the expression of a metabolic biomarker, were deep sequenced and shown to contain mtDNA mutations from the same lineage and progenitor cell [29]. This suggests that the cancer stem cell could be traced solely by mtDNA mutations, independent of any nuclear DNA mutations. However, tumors are functionally heterogeneous and harbor subsets of cancer cells with stem-like features. Consequently, mutations of the mitochondrial genome have been tightly linked to impairment of cellular energy conversion and tissue function [30-32], and further implicated in the pathophysiology of age-associated diseases and aging itself [33, 34].

## Energy sensing mechanisms

Life is a physical system that maintains structure and avoids decay by feeding on negative entropy through metabolism [35]. Changes in metabolites and substrate availability are reflected in the energy output of the whole cell system in the form of the ATP and adenosine monophosphate (AMP) ratio, or other reducing equivalents such as nicotinamide adenine dinucleotide (NAD^+^) vs NADH, which are detected by energy sensing mechanisms. Maintaining a constant ATP level within the cell is crucial, to the extent that all cells maintain a ∆*G*’ATP of approximately -56 kJ/mol [36], and any disruption of this energy balance will compromise cell function and viability [37].

Therefore, one of the central regulators of cellular and organismal metabolism in eukaryotes, and evolutionarily conserved across a multitude of species, is the AMPK [38], which acts as an integrator and mediator of several pathways and processes linking energetics to longevity. AMPK is activated by a high AMP to ATP ratio and then initiates energy producing reactions while inhibiting energy-consuming reactions as a rescue mechanism [38]. In *C. elegans*, changing the catalytic subunit of AMPK by increased expression, led to a lifespan increase of 13% [39], while a constitutively active truncated form of the protein increased life extension by 37.5 % [40]. In mammals, AMPK has a specialized function in metabolically active tissue such as the liver, adipose tissue and muscle, where it acts to integrate nutritional and hormonal signals to food intake, body weight, and substrate homeostasis [41]. AMPK activation is further associated with inhibition of cell proliferation and is an attractive target in cancer treatment, as it shuts off metabolic cell processes needed to maintain cell proliferation [42]. A positive regulator of AMPK is the serine/threonine kinase LKB1, a known tumor suppressor, which phosphorylates the Thr172 in the activation loop of AMPK, thus inducing the downstream effects of AMPK. [43]. The Peutz-Jegher cancer syndrome involves an inherited mutant form of LKB1 and is one of the most commonly known mutations in sporadic human lung cancer [44], and more recently identified in 20% of cervical carcinomas [45]. The loss of LKB1 may therefore facilitate tumor growth under energetically unfavorable conditions. As an example, AMPK activation acutely inhibits fatty acid and cholesterol synthesis through direct phosphorylation of the metabolic enzymes Acetyl-CoA carboxylase (ACC) and HMG-CoA reductase (HMGR) involved in lipid production [46], whereas a defective AMPK sensor system would allow for lipid production even under low energy stress. Increased levels of enzymes such as fatty acid synthase (FASN) involved in cell lipid production have shown to be essential for the survival of a number of cultured tumor cell lines [47-49].

Beyond the lipogenic enzymes, AMPK can acutely modulate glycolysis through phosphorylation of multiple isoforms of phosphofructo-2 kinase (PFK2), a rate-limiting enzyme of glycolysis. PFK2 phosphorylation synthesizes fructose 2,6-bisphosphate, which is a potent stimulator of glycolysis [50], thus increasing the glucose demand. This is seen in response to hypoxic conditions [51], where ATP production from mitochondria drops and AMP levels increase activating AMPK. However, under normoxic conditions, a compensatory increase in mitochondrial volume could strengthen the mitochondrial capacity to produce more ATP. This is supported by findings that AMPK can regulate mitochondrial biogenesis via the p38-PGC-1*α* axis, maintaining cancer cell survival under glucose-limiting normoxic conditions [52]. Getting the cell back on track energetically may be the ultimate goal of AMPK, but also makes this pathway a crucial mediator involved in both cell proliferation and longevity. Metabolic drugs, such as metformin, resveratrol, and 5-aminoimidazole-4-carboxamide-1-D-ribo-furanoside (AICAR), that directly or indirectly activate the AMPK pathway, have been associated with pro-longevity and a reduced risk of developing cancer [53-58].

## Theories of carcinogenesis and Peto’s paradox

Aging and cancer involve many of the same cellular pathways in their progression, suggesting they are closely related in pathology. However, instead of following this lead, there is an ongoing pursuit to define cancers based on largely their mutational patterns. Meanwhile, the heterogenous gene expression pattern found in nearly all cancers begs the question of how critical each individual nuclear gene mutation is in the progression of the disease. Several theories exist to explain carcinogenesis, with the most prevalent being the mutation-centric theory stating that somatic nuclear mutations acquired over time eventually leads to a cancerous transition. Yet, this theory alone is unable to explain why a discrepancy exists between high cancer incidence in small, short-lived animals (i.e. few cells and less mutational events) compared to a low cancer incidence in larger, long-lived animals (i.e. many cells and more mutational events) (visualized in Fig. 1). This observation was named “Peto’s paradox” after the epidemiologist Sir Richard Peto [2]. It highlights the discrepancy that if cancers are initiated by a series of somatic mutations acquired over time and cell number, then organisms with larger body size (more cells) and longer lifespans (more cell divisions) should have a cancer risk that is orders of magnitude greater than organisms of smaller size and shorter lifespan. Thus, in theory, humans should have a higher cancer incidence than mice. However, this is not confirmed in nature, as mice have a higher risk of cancer in their relatively short lifespan (about 4 years) [59], when compared to humans. The mutation-centric theory is established on a belief that there is a linear increase in mutations during cell division as we age. This is in contradiction to data that shows that a substantial portion of somatic mutations (up to 50%) accumulate early in life before full body maturation [1] and seem to slow when stem cells convert from body building to body maintenance [60]. A further challenge with mutagenesis models is the assumption that any mutation can affect cellular fitness, which is not supported by evolutionary theory. From an evolutionary perspective, only mutations that enhance cellular fitness above others in relation to the growth environment will be advantageous [61]. Thus, both mutations and the tissue microenvironment play a role in cell growth. Early-acquired mutations may stay latent and not provide a growth advantage to cells until the tissue microenvironment changes in aging organisms, further enhancing the relationship between aging and cancer risk. This was recently shown by using a computational stochastic model integrating real data on age dependent dynamics of hematopoietic stem cell division. The model demonstrated that previously acquired mutations only became advantageous in an aging microenvironment according to non-cell-autonomous mechanisms [11]. Identification of the most prevalent mutations in cancer and their clear link to metabolic regulations [62] therefore confers that they may be providing the cells with a selective advantage for growth in a changing microenvironment.

## The Warburg effect and relation to Peto’s paradox

**Figure 2.**
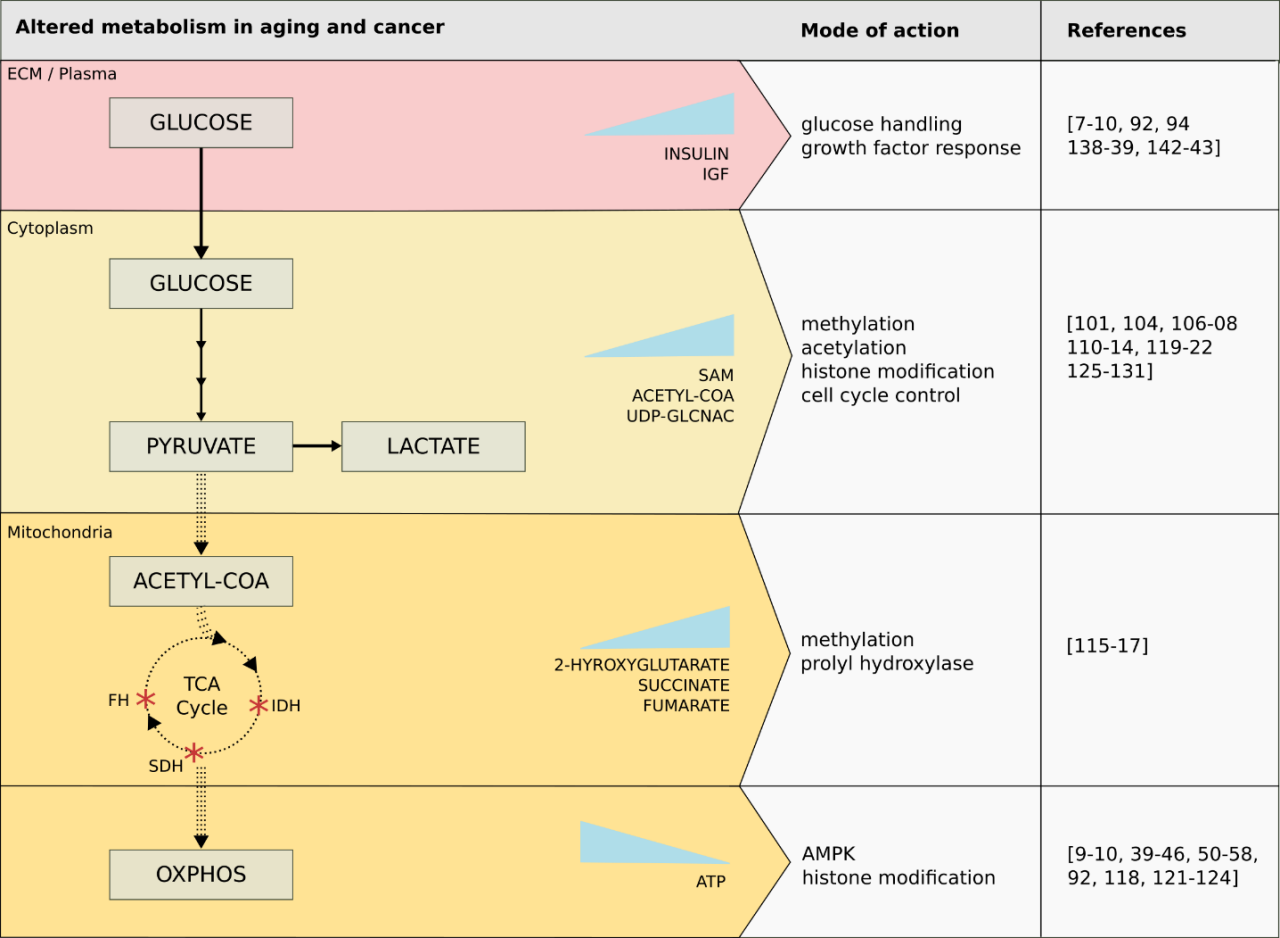
Tumors rarely occur following acute injury to cellular respiration and considerable time is required for non-oxidative energy metabolism (i.e. glycolysis, TCA cycle via substrate-level phosphorylation) to replace oxidative phosphorylation (OXPHOS) as the dominant energy generator of the cell As minor OXPHOS damages accumulated over time, the cell uses substrate-level phosphorylation to compensate gradually for the energy debt. This compensatory effect, by increasing the uptake of glucose and glutamine to be broken down for ATP production, is a well-known hallmark of cancer called “the Warburg effect”. Cells that undergo a Warburg transition and switch their metabolism to glycolysis and glutaminolysis produce increased levels of substrates that can have many downstream effects. Only glucose metabolism is highlighted here, with the solid arrows denoting the increased reliance on glycolysis and production of lactate, and dotted arrows denoting decreased activity in the remainder of the pathway. This translates to lowered production of acetyl-coenzyme-A (acetyl-CoA) from pyruvate, activity of the TCA cycle, and production of precursors necessary to carry out OXPHOS. Also, mutations of key TCA cycle enzymes commonly found in cancer are shown, such as isocitrate dehydrogenase (IDH), succinate dehydrogenase (SDH), and fumarate hydratase (FH), as well as substrates accumulated due to their alterations. Abbreviations: ECM, extracellular matrix; IGF, insulin growth factor; SAM, s-adenosylmethionine; UDP-GlcNAc, uridine diphosphate N-acetylglucosamine; ATP, adenosine triphosphate; AMPK, AMP-activated protein kinase.

Direct signals from mitochondria in the form of substrates, ROS, and other intermediates can affect cellular physiology via genetic and epigenetic mechanisms, and form the foundation for cancer development. The interplay between these metabolic changes, aging, and cancer development is illustrated in Figure 2. In support of the age-associated risk of cancer, tumors rarely occur following acute injury to cellular respiration and considerable time is required for non-oxidative energy metabolism (i.e. glycolysis, TCA cycle via substrate-level phosphorylation) to replace OXPHOS as the dominant energy generator of the cell. Substrate-level phosphorylation can compensate gradually for minor OXPHOS damages accumulated over time. Consequently, expansion of mtDNA mutations affecting ATP production can happen gradually [63] or by asymmetric segregation of cellular content during cell division [12]. This compensatory effect by the continued adaptation by substrate-level phosphorylation for energy production (i.e. increasing the uptake of glucose and glutamine to be broken down for ATP production) is a well-known hallmark of cancer called “the Warburg effect”. Cells that undergo a Warburg transition and switch their metabolism to glycolysis and glutaminolysis produce increased levels of substrates, such as lactate from glycolysis, and succinate, alanine, and aspartate from glutamine or amino acid fermentation [64], that can regulate gene expression epigenetically.

Otto Warburg described the change in cancer metabolism as early as the 1950s [65]. He postulated that the change to this metabolic preference was due to defective mitochondria incapable of producing enough ATP to support cell growth. However, this may not be entirely true as proliferating normal cells, such as activated lymphocytes, also revert to aerobic glycolysis upon growth activation without showing any mitochondrial dysfunction [66]. The current understanding is that rapidly proliferating cells, both normal and cancer, revert to aerobic glycolysis to support the need for new biomass when producing a daughter cell [67]. Thus, in proliferating cells, the metabolic substrate turnover rate increases to support the cell with new cell components. If one considers Warburg’s observations of increased metabolic substrate turnover in cells that are proliferating, it suggests that reduced metabolism should slow cell turnover, be cancer preventative, and halt cell aging. Hence, in relation to Peto’s paradox, larger animals may have evolved a slower metabolic rate that is both cancer preventative and increases longevity. Indeed, the “metabolic rate hypothesis” suggests that cellular metabolic rate and subsequently, oxidative stress, decreases with increasing body size and is protective for larger animals [68]. Slower metabolic rates have been found in large, long-lived animals such as whales and elephants [59, 69-71], compared with smaller animals. A common factor among long-lived animals is that they have no natural enemies and are at reduced risk of predation or death by other external factors, thus the “need for speed” is reduced and more energy can be put into maintaining protective somatic cell maintenance [72, 73].

## Cellular maintenance in an aging system

The influence of metabolism on overall life span is still controversial. However, there must be an evolutionary trade-off to lower metabolic rates in larger animals to avoid cellular harm and increase life span. A possible answer is “somatic maintenance” [74], where the investment in gene repair will increase or decrease to maximize reproductive success in a changing environment. Therefore, lifespans of animals have evolved to maintain fitness and invest in somatic maintenance until reproduction is most likely to be achieved. This explains why most wild animals do not develop cancer, as they do not survive beyond reproductive age in the wild. Humans are the only species that have developed technological advances and designed their living environment to extend their lifespan far beyond reproductive age, thus exposing themselves to a variety of diseases associated with old age. This also holds true for animals kept in captivity, which live beyond their normal lifespan in the wild, and do develop cancer [75, 76].

The low cancer incidence in larger species could also relate to cell size. The difference in cell size among species has been well documented, with blood cell sizes ranging from 78 to 110 to 170 and 215 µm^2^ in shrews, rats, humans, and whales, respectively [77, 78]. If cell size is considered when modeling cancer rates between species, then this skewed cancer risk almost disappears [68]. More importantly, in addition to the association of drop in cancer rate, larger cells exhibit a lower metabolic rate [79] and prioritize slow growth over division [80]. Furthermore, cell volume and metabolic rate scale with body mass in 13 different cell types [81], supporting the model in which metabolic rate and somatic maintenance play a crucial role in cancer development [82]. This phenomenon is only found between species and not within, as obese humans have an increased cancer risk [83] (Fig. 1). Nature’s way of combating the inherent risk of living larger is exemplified in elephants which have an increased copy number of the tumor suppressor P53 [84], leading to an improved gene maintenance system with reduced cancer risk and a longer lifespan [69]. Some exceptions to the rule do exist. For example, the naked mole rat - a small, subterranean rodent - has been found to live over 30 years, while exhibiting no documented cases of cancer [85]. As with elephants, it was recently found that the naked mole rat had fibroblasts producing a type of high-molecular-mass glycosaminoglycan, which increase extracellular matrix tension and further signals to a cell cycle checkpoint p16INK4a that inhibits cell cycle progression [86]; thus supporting the notion that the tissue microenvironment plays a major role in cell cycle regulation.

Since continual growth and aging seem to be linked, with aging perhaps being directed by this unnecessary growth [87], evidence points to energy partitioning away from biogenesis and to cell maintenance as a way to extend lifespan [88]. Cellular maintenance is carried out in various ways and exists to combat the inherent mistakes our cells accumulate as they age. The main contributors of these are gene and protein errors, and while gene instability is hallmark of cancer, we are exploring factors beyond genetics in aging and cancer. Protein homeostasis (proteostasis) is vital for quality control of the cell proteome in an aging system. Altered proteostasis can occur upon the accumulation of dysfunctional proteins due to mutations and misfolded of proteins from lack of necessary enzymes or chaperones, incorrect compartmentalization, and problems in degradation systems for the clearing of these proteins, such as autophagy-lysosomal and ubiquitin-proteasome systems [89]. Cell-stress-signaling pathways regulate the proteostasis network and prevent the toxicity associated with misfolded proteins that could aggregate in subcellular compartments and tissues. The efficiency of the proteostasis network declines with age and this failure in protein homeostasis has been proposed to underlie the basis of common age-related human disorders [90].

Autophagy is a major driver of this housekeeping role whereby unwanted, excess, or damaged cytosolic components are self-degraded by the cell through lysosomal digestion. Autophagy is one of the programmed self-degradative processes that is important for balancing sources of energy at critical times in development and in response to cellular stress [91]. Many pathways are in place to detect nutrient stress (AMPK, mTOR, FOXO), hypoxia (HIF), misfolded proteins (unfolded protein response), immune response (NF-kB, MAPK), DNA damage (P53), mitochondrial stress (MMP, PINK) [92]. The selective removal of damaged mitochondria in particular has been termed mitophagy and dysregulation of this clearance is a risk factor for cancer development [93]. With decreased mitophagy, a slow accumulation of dysfunctional mitochondria may lead to accumulation of metabolic substrates causing epigenetic signaling changes and altered gene expression. In lifestyle diseases such as obesity and type 2 diabetes [94], dysfunctional mitochondria may not be cleared due to a constant excess of nutrient availability inhibiting the autophagic response mechanisms. Meanwhile, the strengthening of other metabolic pathways such as glycolysis to compensate for energy deficit provides the foundation for cancer cell transformation [95]. In mammals, one of AMPK’s many targets is the UNC-51-like kinase 1 (ULK1), which regulates the formation of the autophagosome in response to energetic stress. This regulation is thought to be an important mediator of organismal aging [96]. Most longevity-promoting interventions require an intact autophagic machinery; furthermore, reduced autophagic activity is associated with aging while evidence suggests that enhanced autophagy promotes longevity and delays age-related phenotypes [97].

## Metabolism and epigenetic changes drive aging and cancer

Deregulation and loss of homeostasis is a driver of damage and dysfunction frequently encountered in aging and cancer, affecting all functions of the cell. Normal systemic operation requires the proper flexibility and functioning of gene and protein expression to respond adequately to intracellular and extracellular signals. This flexibility is made possible by epigenetic control over how the primary DNA sequence is expressed. Besides changes in bioenergetics and creation of cellular building blocks, metabolism can affect function through control of gene expression. Much of the enzymatic maintenance of epigenetic patterns occurs through information provided by metabolic substrates and metabolites, signaling when to grow and trigger transcription based on nutrient availability. The crucial role they hold in the support of cancer growth has been proven in elegant experiments in which enucleated normal cells were fused with nuclei from tumor cells to form cybrids. These cybrid cells did not form malignant cells, whereas tumor cells infused with normal cytoplasm had their tumorigenic potential in mice reduced from 92% to 51% [98]. The important feature of their study was that the non-transformed and transformed cells all originated from a cloned progenitor cell with a common nuclear and cytoplasmic background. When the experiments were conducted, the authors did not identify the nature of the observed effects, but with experimental results from a more recent study focusing on the role of mitochondria in this situation [99], it is highly likely that substrates and intermediates of metabolism could drive this effect via epigenetic regulation. These experiments and the increasing data showing that most of the common mutations found in cancers are related to changes in metabolism [62], demonstrate that there is more than just gene mutations driving cancer and aging. Compounds directly essential to the function of epigenetic enzymes are produced during metabolism, such as S-adenosylmethionine (SAM) and alpha-ketoglutarate (α-KG), nicotinamide adenine dinucleotide (NAD) and acetyl coenzyme-A (acetyl-CoA), and uridine diphosphate N-acetylglucosamine (UDP-GlcNAc). ATP is also vital for proper function but not rate limiting for these enzymatic reactions due to its relative abundance [100].

### Methylation

SAM is produced in the cell from glycolytic intermediates shuttled to serine metabolism [101] and by addition of adenosyl to methionine from ATP. Histone methyltransferases (HMTs) and DNA methyltransferases (DNMTs) both use SAM as a methyl donor for transfer to the 5’ carbon on cytosine, lysine or arginine residues of histones, respectively. α-KG is formed both in the TCA cycle from glucose-derived isocitrate by isocitrate dehydrogenase (IDH) and by transamination of glutamate [102] and is essential to the demethylation functions of lysine-specific histone demethylase 1 (LSD1) and JmjC-domain containing histone demethylase (JHDM) on histones, and ten-eleven translocation (TET) demethylase on DNA [103]. The effect of histone methylation depends on the specific proteins modified, and can have either repressive or enhancing effects on transcription [104], while DNA methylation results in reduced expression. There is a documented trend of methylation changes in aging, termed epigenetic drift [105], but it is difficult to tie it with specific functional implications absent of pathological symptoms. However, in a differential analysis of methylation in islet cells, genes associated with mitochondrial function and diabetes are targeted in this aging phenomenon [106]. In a study of 58 cancer cell types, DNA enhancer methylation was a strong predictor of cancer-related gene expression and of the 207 of hypomethylated/upregulated genes, two-thirds had function in metabolic processes [107]. Conversely, reduced expression of IDH and TET have been associated with decreased survival in chronic lymphocytic leukemia (CLL), but did not correlate with any measured change in global methylation [108].

### Acetylation

One of the clearest links between metabolism and epigenetics is through acetyl-CoA. Acetyl-CoA plays a major role in cellular nutrient sensing is generated during oxidation of pyruvate and fatty acids in the mitochondria and from citrate in the cytosol and nucleus [109], and also serves as a substrate for lipogenesis in the cytosol. Acetyl-CoA levels directly affect the activity of histone acetyltransferases (HAT) as acetyl donors, and can act indirectly on histone deacetylation by Sirtuins due to its role in modulating NAD^+^/NADH by availability to the TCA cycle [110]. Histone acetylation and demethylation generally result in an open structure allowing for expression of the region, while deacetylation produces a tighter chromatin structure and reduced expression [111]. Acetyl-CoA is dynamically regulated by glucose availability in cancer cells and the ratio of acetyl-CoA: coenzyme A within the nucleus modulates global histone acetylation levels. Reduced sirtuin (SIRT7) activity (increased acetylation) is associated with stem cell senescence and mitochondrial unfolded protein response [112]. Additionally, expression of *ATG* autophagy genes are tightly controlled by acetylation and thus dependent upon acetyl-coA levels and nutrient sensing by the cell with the reduction of acetyl-coA synthesis, promoting autophagy and extending lifespan in drosophila [113]. Testing *in vitro* and confirmation *in vivo* has shown histone acetylation to be controlled by glucose availability and AKT-activation of ATP-citrate lyase (ACLY), the enzyme responsible for production of acetyl-CoA from citrate [114].

### TCA Cycle Intermediates

In the TCA cycle, metabolites such as succinate and fumarate are essential for completion of the cycle, but they may also accumulate due to altered expression of succinate dehydrogenase (SDH) and fumarate hydratase (FH) inhibiting prolyl hydroxylases (PHD) and increasing HIF levels [115]. IDH mutants produce 2-hydroxyglutarate, similar in structure to α-KG, which interferes with the normal TCA cycle and other enzymatic reactions, such as demethylation, that depend on α-KG as a substrate [116]. Mutations in IDH, FH, and SDH are common in cancer and these metabolites contribute to cancer growth and survival by reduced expression of tumor suppression genes [117].

### Chromatin modifications

Indirectly, modifications of enzymes responsible for these epigenetic changes can increase or decrease their activity. This occurs mainly through phosphorylation and O-linked N-acetylglucosamine (O-GlcNAc) glycosylation. Modification of histones can occur by the activity of AMPK through phosphorylation of histone protein H2B, resulting in expression of genes important to the cellular energy homeostasis [118]. Changes in histones by glycosylation (formation of O-GlcNAc) are catalyzed by O-GlcNAc transferase, but little is established on the function or consequence of this histone modification type. Recently, it has been shown to have a role in H3.3-histone cell cycle regulator mediated nucleosome assembly for transcription and also cellular senescence through its regulation of chromatin dynamics [119]. The O-GlcNAc glycosylations are directly affected by nutrient availability signals and activity is reliant upon UDP-GlcNAc as a donor substrate, a product of the hexosamine biosynthesis pathway (fed by 2-5% of imported glucose), upregulated in cancer [120]. Modification of histones through phosphorylation and O-GlcNAc glycosylation are implicated in cell cycle control [121, 122] with the two exhibiting inverse relationships during different cell cycle phases. Phosphorylation of histones has been found to be a significant marker of tumor grade and mitotic index in breast cancer [123] and proliferative marker in bladder cancer [124]. Increased O-GlcNAc glycosylation is consistently found in cancer as well (breast [125, 126], prostate [127], lung [128], colorectal [128, 129], liver [130], and nonsolid cancers such as chronic lymphocytic leukemia [131]) and has been correlated with increased metastatic potential. Unique modifications of histones for degradation also reveal the importance of histone turnover and homeostasis (and proteostasis) in epigenetic regulation [132]. In aging cells with DNA-damage signal activation, histone synthesis is reduced, demonstrating other ways in which histone control can affect cell homeostasis [133].

## Lifestyle modulates longevity and cancer development

Cellular dysfunction and stress are recurring themes presented in this review for their likely role in accelerating aging and induction of cancer. This is largely due to increased metabolic activity, abnormal metabolism through diet or genetic/epigenetic modification, and inhibition of healthy cellular maintenance. These conditions are all capable of improvement through lifestyles changes.

In the developed world, food sources are constantly available and reduction of metabolic activity by prolonged fasting is rarely achieved. We no longer experience seasonal or periodic fluctuations in nutrient availability like our ancestors, but still have a vital and complex nutrient sensing system that can be severely affected by our modern diets. Reducing this excess consumed energy should result in a longer and healthier lifespan by decreasing metabolic activity and energy partitioning, ROS, and epigenetic-affecting metabolites. Calorie restriction was one of the first diets to show a direct relationship between metabolism and lifespan extension [134], and continues to be lauded as the best option for life extension and health [135]. The consumption of reduced calories, but not below nutritional levels, has been shown to reduce resting metabolic rate [136], and depends on mitochondrial function for its beneficial outcomes [137]. Calorie restriction and associated dietary restriction seem to exert their effects specifically through mTOR, AMPK, and glucose handling (IGF/insulin) pathways [138, 139] with outcomes such as lifespan extension, reduced inflammation and cancer. Taking the restriction further to a pure ketogenic diet has been shown in mice to reduce metabolic activity and increase uncoupling protein 2 and the ketone, beta-hydroxybutyrate [140]. Ketone bodies are important compounds in the body’s response to restricted nutrients; as a precursor to acetyl-CoA it serves as an energy source and donor for epigenetic modifications [141]. For more information on lifestyle modulations such as calorie and dietary restrictions, micro/macronutrients, and meal timing, Fontana *et al.* [142] provides a complete review. Diet is not the only negative modern lifestyle factor we have altered, however; sleep and circadian alignment are adjusted to fit societal norms and they also have large effects on metabolism and cell recovery. Most research in this area is performed on shift workers or through short-term sleep deprivation. While only shift work results in circadian misalignment, even a single night of sleep restriction can mimic its effects and both have been related to increased insulin resistance [143, 144].

Calorie and dietary restrictions in combination with regular sleeping patterns can lead to a more normal metabolism primarily through direct influx of nutrients and also regulation of cellular processes through healthy maintenance. The mild stress on mitochondria by preserving short periods of low-nutrients can induce retrograde stress responses and actually enhance cellular function by retaining the activity of autophagic processes. As mentioned before in relation to obesity and diabetes II, such activation of unfolded protein response and IGF has been induced by redox stress to mitochondria (mitohormesis) in muscle of Drosophila, resulting in increased mitophagy and lifespan [94]. These changes in lifestyle have persistent effects in major metabolic pathways, ROS, mitochondrial turnover, immune regulation, epigenetic control, and DNA repair; related to both aging and cancer.

## Concluding remarks

Further investigation into the differentiating genetic or metabolic factors between species is a key to understanding the source of neoplasms and the mechanisms nature has adapted to fight them. With the explanations given here and even the outliers of Peto’s paradox, a need to explain cancer formation using only the multi-stage mutational model lessens. The refitting of the model with respect to cell size and volume, differential tissue behavior, and the presence of long-living organisms with no cancer at all could have a unifying relationship within cell metabolism. Larger cells, less proliferating tissues, and long-living organisms all have slow metabolism and consequently slower cell growth. Upon analysis, carcinogenesis may be driven by a forced change in metabolism that is not tolerated by an organism or tissue and its adapted mechanisms.

To further expand the knowledge of how these factors are regulating aging and cancer development, there is a need to share and compare the accumulated evidence that underlies these mechanisms. The close relationship between aging, cancer, and metabolism across species can be addressed using accessible databases depositing published data. The era of computer science and technology which in many aspects seem to be outpacing our evolutionary adaptation to lifestyle changes, could very well be the technology that will help solve this puzzle. The ability to completely prevent aging and cancer is doubtful, but as said by Stauch *et al.*, “Aging is not necessarily pathogenic, and in healthy aging, organs, cells and subcellular organelles can respond to gradual age-associated stress’’ [145]. Armed with knowledge that our diet and behavior matter on a molecular level, we can make healthy lifestyle choices and allow our bodies to combat this gradual stress, while attempting to alleviate the modern prevalence of aging pathology and tumorigenesis.
